# Supplementary material for: A bacterial immunity protein directly senses two disparate phage proteins
Source: Nature. 2024 Oct 16;635(8039):728–35. doi: 10.1038/s41586-024-08039-y (PMC11578894; doi:10.1038/s41586-024-08039-y)
Supplement: Supplementary file 1 — This file contains Supplementary Fig. 1 and Tables 1–3. [file 41586_2024_8039_MOESM1_ESM.pdf]

nature portfolio

https://doi.org/10.1038/s41586-024-08039-y

Supplementary information

A bacterial immunity protein directly senses two disparate phage proteins

In the format provided by the authors and unedited

Supplementary Figure 1: raw gels

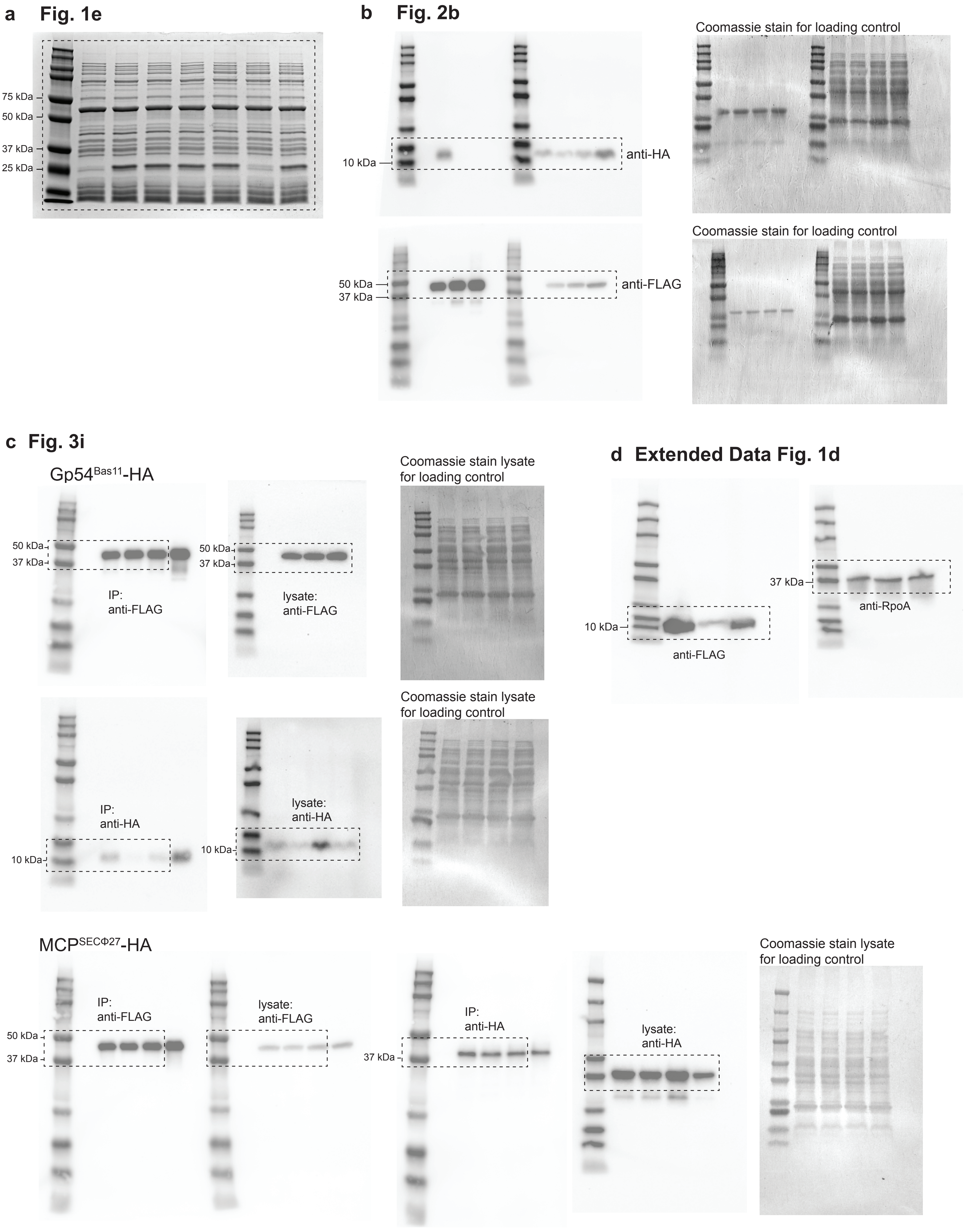

SI Table 1. Strains

Bacterial Strains

Name	Genotype	Source
ML6	MG1655	
	DH5α	Invitrogen
ML3840	MG1655 pBR322-EV	Zhang et al. 2022
ML3841	MG1655 pBR322-*capRel*^{SJ46}	Zhang et al. 2022
ML3857	MG1655 pBR322-*capRel*^{SJ46} pBAD33-EV	Zhang et al. 2022
ML4237	MG1655 pBR322-*capRel*^{SJ46} pBAD33-*gp54*^{Bas11}	this study
ML4238	MG1655 pBR322-*capRel*^{SJ46} pBAD33-*gp54*^{Bas11}*(W43*)*	this study
ML4239	MG1655 pBR322-*capRel*^{SJ46} pBAD33-*gp54*^{Bas11}*(G24D)*	this study
ML4240	BL21(DE3) pET-His_6-*gp54*^{Bas11}	this study
ML4241	BL21(DE3) pET-His_6-*gp54*^{Bas11}*(G24D)*	this study
ML4242	BL21(DE3) pET-His_6-*MBP-capRel*^{SJ46}	this study
ML4243	MG1655 pBR322-*capRel*^{SJ46} pBAD33-*gp54*^{Bas11}*-HA*	this study
ML4244	MG1655 pBR322-*capRel*^{SJ46}*-FLAG* pBAD33-*gp54*^{Bas11}*-HA*	this study
ML4245	MG1655 pBR322-*capRel*^{SJ46}*-FLAG* pBAD33-*gp54*^{Bas11}*(G24D)-HA*	this study
ML4246	MG1655 pBR322-*capRel-chimera-FLAG* pBAD33-*gp54*^{Bas11}*-HA*	this study
ML3842	MG1655 pBR322-*capRel*^{Ebc}	Zhang et al. 2022
ML3846	MG1655 pBR322-*capRel-chimera*	Zhang et al. 2022
ML4247	MG1655 pBR322-*capRel-chimera* pBAD33-*gp54*^{Bas11}	this study
ML4248	MG1655 pBR322-EV pBAD33-*gp54*^{Bas11}	this study
ML4249	MG1655 pBR322-*capRel*^{SJ46}*(L270P)* pBAD33-*gp54*^{Bas11}	this study
ML4250	MG1655 pBR322-*capRel*^{SJ46}*(N275D)* pBAD33-*gp54*^{Bas11}	this study
ML4251	MG1655 pBR322-*capRel*^{SJ46}*(L276P)* pBAD33-*gp54*^{Bas11}	this study
ML4252	MG1655 pBR322-*capRel*^{SJ46}*(D273K)* pBAD33-*gp54*^{Bas11}	this study
ML4253	MG1655 pBR322-*capRel*^{SJ46}*(K278E)* pBAD33-*gp54*^{Bas11}	this study
ML4254	MG1655 pBR322-*capRel*^{SJ46}*(S279P)* pBAD33-*gp54*^{Bas11}	this study
ML4255	MG1655 pBR322-*capRel*^{SJ46}*(K278E R314E K316E)* pBAD33-*gp54*^{Bas11}	this study
ML3865	MG1655 pBR322-EV pBAD33-*mcp*$^{SEC\Phi27}$	Zhang et al. 2022
ML3858	MG1655 pBR322-*capRel*^{SJ46} pBAD33-*mcp*$^{SEC\Phi27}$	Zhang et al. 2022
ML4256	MG1655 pBR322-*capRel*^{SJ46}*(N275D)* pBAD33-*mcp*$^{SEC\Phi27}$	this study
ML4257	MG1655 pBR322-*capRel*^{SJ46}*(K278E)* pBAD33-*mcp*$^{SEC\Phi27}$	this study

ML4258	MG1655 pBR322-$capRel^{SJ46}(K278E\ R314E\ K316E)$ pBAD33-$mcp^{SEC\Phi27}$	this study
ML4259	MG1655 pBR322-$capRel^{SJ46}(N275D)$-$FLAG$ pBAD33-$gp54^{Bas11}$-HA	this study
ML4260	MG1655 pBR322-$capRel^{SJ46}(K278E)$-$FLAG$ pBAD33-$gp54^{Bas11}$-HA	this study
ML3861	MG1655 pBR322-$capRel^{SJ46}$ pBAD33-$mcp^{SEC\Phi27}$-HA	Zhang et al. 2022
ML3862	MG1655 pBR322-$capRel^{SJ46}$-$FLAG$ pBAD33-$mcp^{SEC\Phi27}$-HA	Zhang et al. 2022
ML4261	MG1655 pBR322-$capRel^{SJ46}(N275D)$-$FLAG$ pBAD33-$mcp^{SEC\Phi27}$-HA	this study
ML4262	MG1655 pBR322-$capRel^{SJ46}(K278E)$-$FLAG$ pBAD33-$mcp^{SEC\Phi27}$-HA	this study
ML4263	MG1655 pBR322-$capRel^{SJ46}$ pBAD33-mcp^{Bas10}	this study
ML4264	MG1655 pBR322-$capRel^{SJ46}$ pBAD33-$mcp^{Bas10}(I115F)$	this study
ML4265	MG1655 pBR322-$capRel^{SJ46}$ pBAD33-$gp57^{Bas10}$	this study
ML4266	MG1655 pBR322-$capRel^{SJ46}$ pBAD33-$gp60^{Bas8}$	this study
ML4267	MG1655 pBR322-$capRel^{SJ46}$ pBAD33-$gp57^{Bas5}$	this study
ML4268	MG1655 pBR322-$capRel^{SJ46}$ pBAD33-$gp19^{SEC\Phi27}$	this study
ML4269	MG1655 pBR322-$capRel^{SJ46}$ pBAD33-$gp54^{Bas11}(D6E)$	this study
ML4270	MG1655 pBR322-$capRel^{SJ46}$ pBAD33-$gp54^{Bas11}(A7V)$	this study
ML4271	MG1655 pBR322-$capRel^{SJ46}$ pBAD33-$gp54^{Bas11}(I25V)$	this study
ML3881	MG1655 pBR322-EV pBAD33-EV	Zhang et al. 2022
ML4272	MG1655 pBR322-EV pBAD33-$gp54^{Bas11}(W43*)$	this study
ML4273	MG1655 pBR322-EV pBAD33-$gp54^{Bas11}(G24D)$	this study
ML4274	MG1655 pBR322-$capRel^{SJ46}(L270P)$	this study
ML4275	MG1655 pBR322-$capRel^{SJ46}(N275D)$	this study
ML4276	MG1655 pBR322-$capRel^{SJ46}(L276P)$	this study
ML4277	MG1655 pBR322-$capRel^{SJ46}$ pBAD33-$gp57^{Bas10}(V7A)$	this study
ML4278	MG1655 pBR322-$capRel^{SJ46}$ pBAD33-$gp57^{Bas10}(V25I)$	this study
ML4279	MG1655 pBR322-$capRel^{SJ46}$ pBAD33-$gp57^{Bas10}(V7A\ V25I)$	this study
ML4280	MG1655 pBR322-$capRel^{SJ46}$ pBAD33-$gp54^{Bas11}(D6G)$	this study
ML4281	MG1655 pBR322-$capRel^{SJ46}$ pBAD33-$gp54^{Bas11}(S39C)$	this study
ML4282	MG1655 pBR322-$capRel^{SJ46}$ pBAD33-$gp54^{Bas11}(L41P)$	this study
ML3897	DH5α pBAD33-EV	Zhang et al. 2022
ML3901	DH5α pBR322-EV	Zhang et al. 2022
ML3902	DH5α pBR322-$capRel^{SJ46}$	Zhang et al. 2022
ML3904	DH5α pBR322-$capRel^{Ebc}$	Zhang et al. 2022
ML3907	DH5α pBR322-*capRel-chimera*	Zhang et al. 2022

ML4283	DH5α pBAD33-*gp54*^{Bas11}	this study
ML4284	DH5α pBAD33-*gp54*^{Bas11}*(W43*)*	this study
ML4285	DH5α pBAD33-*gp54*^{Bas11}*(G24D)*	this study
ML4286	DH5α pET-*His*$_{6}$-*gp54*^{Bas11}	this study
ML4287	DH5α pET-*His*$_{6}$-*gp54*^{Bas11}*(G24D)*	this study
ML4288	DH5α pET-*His*$_{6}$-*MBP-capRel*^{SJ46}	this study
ML4289	DH5α pBAD33-*gp54*^{Bas11}*-HA*	this study
ML3921	DH5α pBR322-*capRel*^{SJ46}*-FLAG*	Zhang et al. 2022
ML4290	DH5α pBAD33-*gp54*^{Bas11}*(G24D)-HA*	this study
ML3961	DH5α pBR322-*capRel-chimera-FLAG*	Zhang et al. 2022
ML4291	DH5α pBR322-*capRel*^{SJ46}*(L270P)*	this study
ML4292	DH5α pBR322-*capRel*^{SJ46}*(N275D)*	this study
ML4293	DH5α pBR322-*capRel*^{SJ46}*(L276P)*	this study
ML4294	DH5α pBR322-*capRel*^{SJ46}*(D273K)*	this study
ML4295	DH5α pBR322-*capRel*^{SJ46}*(K278E)*	this study
ML4296	DH5α pBR322-*capRel*^{SJ46}*(S279P)*	this study
ML4297	DH5α pBR322-*capRel*^{SJ46}*(K278E R314E K316E)*	this study
ML3918	DH5α pBAD33-*mcp*$^{SEC\Phi27}$	Zhang et al. 2022
ML4298	DH5α pBR322-*capRel*^{SJ46}*(N275D)-FLAG*	this study
ML4299	DH5α pBR322-*capRel*^{SJ46}*(K278E)-FLAG*	this study
ML3922	DH5α pBAD33-*mcp*$^{SEC\Phi27}$*-HA*	Zhang et al. 2022
ML4300	DH5α pBAD33-*mcp*^{Bas10}	this study
ML4301	DH5α pBAD33-*mcp*^{Bas10}*(I115F)*	this study
ML4302	DH5α pBAD33-*gp57*^{Bas10}	this study
ML4303	DH5α pBAD33-*gp60*^{Bas8}	this study
ML4304	DH5α pBAD33-*gp57*^{Bas5}	this study
ML4305	DH5α pBAD33-*gp19*$^{SEC\Phi27}$	this study
ML4306	DH5α pBAD33-*gp54*^{Bas11}*(D6E)*	this study
ML4307	DH5α pBAD33-*gp54*^{Bas11}*(A7V)*	this study
ML4308	DH5α pBAD33-*gp54*^{Bas11}*(I25V)*	this study
ML4309	DH5α pBAD33-*gp57*^{Bas10}*(V7A)*	this study
ML4310	DH5α pBAD33-*gp57*^{Bas10}*(V25I)*	this study
ML4311	DH5α pBAD33-*gp57*^{Bas10}*(V7A V25I)*	this study

ML4312	DH5α pBAD33-*gp54*^{Bas11}*(D6G)*	this study
ML4313	DH5α pBAD33-*gp54*^{Bas11}*(S39C)*	this study
ML4314	DH5α pBAD33-*gp54*^{Bas11}*(L41P)*	this study
ML4316	MG1655 pBR322-*gp54*^{Bas11}*-FLAG*	this study
ML4317	MG1655 pBR322-*gp54*^{Bas11}*-FLAG(-37mut)*	this study
ML4318	MG1655 pBR322-*gp54*^{Bas11}*-FLAG(-71mut)*	this study

Phage Strains

**Name**	**Genotype**	**Source**
phML6	T7	Zhang et al. 2022
phML11	SECΦ27	Doron et al. 2018
phML80	Bas11	Maffei et al. 2021
phML81	Bas11 escape clone 1	this study
phML82	Bas11 escape clone 2	this study
phML83	Bas11 escape clone 3	this study
phML84	Bas11 escape clone 4	this study
phML85	Bas11 escape clone 5	this study
phML86	Bas11 escape clone 6	this study
phML87	Bas10	Maffei et al. 2021
phML88	Bas10 escape clone 1	this study
phML89	Bas10 escape clone 2	this study
phML90	Bas11 *gp54* deletion evolved clone 1	this study
phML91	Bas11 *gp54* deletion evolved clone 2	this study
phML92	Bas11 containing *mcp*^{Bas11}*(I115F)*	this study
phML43	SECΦ27 containing *mcp*$^{SEC\Phi27}$*(L114P)*	Zhang et al. 2022
phML93	SECΦ27 containing *gp54*^{Bas11}*mcp*$^{SEC\Phi27}$*(L114P)*	this study
phML94	SECΦ27 containing *gp54*^{Bas11}	this study

SI Table 2. Plasmids

**Plasmid**	**Description**	**Source**
pBAD33-EV	empty vector of pBAD33 (p15A ori, P_{ara} promoter)	lab stock
pBR322-EV	derivative of pBR322 with P_{tet} removed	lab stock
pBR322-*capRel*^{SJ46}	*capRel*^{SJ46} with native promoter	Zhang et al. 2022
pBR322-*capRel*^{Ebc}	*capRel*^{Ebc} with native promoter	Zhang et al. 2022
pBR322-*capRel-chimera*	*capRel* chimera producing CapRel^{SJ46} (272-341) replaced with CapRel^{Ebc} (270-339)	Zhang et al. 2022
pBAD33-*gp54*^{Bas11}	arabinose inducible *gp54*^{Bas11}	this study
pBAD33-*gp54*^{Bas11}*(W43*)*	arabinose inducible *gp54*^{Bas11}*(W43*)*	this study
pBAD33-*gp54*^{Bas11}*(G24D)*	arabinose inducible *gp54*^{Bas11}*(G24D)*	this study
pET-*His*$_6$-*gp54*^{Bas11}	*gp54*^{Bas11} with N-terminal *His*$_6$*-tag* under T7 promoter	this study
pET-*His*$_6$-*gp54*^{Bas11}*(G24D)*	*gp54*^{Bas11}*(G24D)* with N-terminal *His*$_6$*-tag* under T7 promoter	this study
pET-*His*$_6$*-MBP-capRel*^{SJ46}	*His*$_6$*-MBP-capRel*^{SJ46} under T7 promoter	this study
pBAD33-*gp54*^{Bas11}*-HA*	arabinose inducible *gp54*^{Bas11} with C-terminal *HA-tag*	this study
pBR322-*capRel*^{SJ46}*-FLAG*	*capRel*^{SJ46} with C-terminal *FLAG-tag*	Zhang et al. 2022
pBAD33-*gp54*^{Bas11}*(G24D)-HA*	arabinose inducible *gp54*^{Bas11}*(G24D)* with C-terminal *HA-tag*	this study
pBR322-*capRel-chimera-FLAG*	*Chimera* with C-terminal *FLAG-tag*	Zhang et al. 2022
pBR322-*capRel*^{SJ46}*(L270P)*	*capRel*^{SJ46}*(L270P)* with native promoter	this study
pBR322-*capRel*^{SJ46}*(N275D)*	*capRel*^{SJ46}*(N275D)* with native promoter	this study
pBR322-*capRel*^{SJ46}*(L276P)*	*capRel*^{SJ46}*(L276P)* with native promoter	this study
pBR322-*capRel*^{SJ46}*(D273K)*	*capRel*^{SJ46}*(D273K)* with native promoter	this study
pBR322-*capRel*^{SJ46}*(K278E)*	*capRel*^{SJ46}*(K278E)* with native promoter	this study
pBR322-*capRel*^{SJ46}*(S279P)*	*capRel*^{SJ46}*(S279P)* with native promoter	this study
pBR322-*capRel*^{SJ46}*(K278E R314E K316E)*	*capRel*^{SJ46}*(K278E R314E K316E)* with native promoter	this study
pBAD33-*mcp*$^{SEC\Phi27}$	arabinose inducible *mcp*$^{SEC\Phi27}$	Zhang et al. 2022
pBR322-*capRel*^{SJ46}*(N275D)-FLAG*	*capRel*^{SJ46}*(N275D)* with C-terminal *FLAG-tag*	this study
pBR322-*capRel*^{SJ46}*(K278E)-FLAG*	*capRel*^{SJ46}*(K278E)* with C-terminal *FLAG-tag*	this study
pBAD33-*mcp*$^{SEC\Phi27}$*-HA*	arabinose inducible *mcp*$^{SEC\Phi27}$ with C-terminal *HA-tag*	Zhang et al. 2022

pBAD33-*mcp*^{Bas10}	arabinose inducible *mcp*^{Bas10}	this study
pBAD33-*mcp*^{Bas10}*(I115F)*	arabinose inducible *mcp*^{Bas10}*(I115F)*	this study
pBAD33-*gp57*^{Bas10}	arabinose inducible *gp57*^{Bas10} *(gp54*^{Bas11} *homolog)*	this study
pBAD33-*gp60*^{Bas8}	arabinose inducible *gp60*^{Bas8}*(gp54*^{Bas11} *homolog)*	this study
pBAD33-*gp57*^{Bas5}	arabinose inducible *gp57*^{Bas5} *(gp54*^{Bas11} *homolog)*	this study
pBAD33-*gp19*$^{SEC\Phi27}$	arabinose inducible *gp19*$^{SEC\Phi27}$*(gp54*^{Bas11} *homolog)*	this study
pBAD33-*gp54*^{Bas11}*(D6E)*	arabinose inducible *gp54*^{Bas11}*(D6E)*	this study
pBAD33-*gp54*^{Bas11}*(A7V)*	arabinose inducible *gp54*^{Bas11}*(A7V)*	this study
pBAD33-*gp54*^{Bas11}*(I25V)*	arabinose inducible *gp54*^{Bas11}*(I25V)*	this study
pBAD33-*gp57*^{Bas10}*(V7A)*	arabinose inducible *gp57*^{Bas10}*(V7A)*	this study
pBAD33-*gp57*^{Bas10}*(V25I)*	arabinose inducible *gp57*^{Bas10}*(V25I)*	this study
pBAD33-*gp57*^{Bas10}*(V7A V25I)*	arabinose inducible *gp57*^{Bas10}*(V7A V25I)*	this study
pBAD33-*gp54*^{Bas11}*(D6G)*	arabinose inducible *gp54*^{Bas11}*(D6G)*	this study
pBAD33-*gp54*^{Bas11}*(S39C)*	arabinose inducible *gp54*^{Bas11}*(S39C)*	this study
pBAD33-*gp54*^{Bas11}*(L41P)*	arabinose inducible *gp54*^{Bas11}*(L41P)*	this study
pBR322-*gp54*^{Bas11}*-FLAG*	*gp54*^{Bas11} with C-terminal *FLAG-tag* and wild-type native promoter	this study
pBR322-*gp54*^{Bas11}*-FLAG(-37mut)*	*gp54*^{Bas11} with C-terminal *FLAG-tag* and native promoter from escape clone 4	this study
pBR322-*gp54*^{Bas11}*-FLAG(-71mut)*	*gp54*^{Bas11} with C-terminal *FLAG-tag* and native promoter from escape clone 3	this study

SI Table 3. Primers

Name	Purpose	Sequence (5'-3')
TZ-1	linearize pBAD33	AAGCTTGGCTGTTTTGGC
TZ-2	linearize pBAD33	CTCGAATTCGCTAGCCCAAA
TZ-3	*gp54*^{Bas11} into pBAD33	TACCCGTTTTTTTGGGCTAGCGAATTCGAGAT GCAAGACCAGATAGACGCT
TZ-4	*gp54*^{Bas11} into pBAD33	CTTCTCTCATCCGCCAAAACAGCCAAGCTTTT AAATAACCTCCATAACAGTATGCTCGT
TZ-5	linearize pET-His_6	GCTGCCGTGATGGTGATGGTGATGTTTCATGG TATA
TZ-6	linearize pET-His_6	AAGGGTGGGCGCGCCG
TZ-7	*gp54*^{Bas11} into pET-His_6	ACATCACCATCACCATCACGGCAGCAGCGGC ATGCAAGACCAGATAGACGCT
TZ-8	*gp54*^{Bas11} into pET-His_6	CTGGGTCGGCGCGCCCACCCTTTTAAATAACC TCCATAACAGTATGCTCGT
TZ-9	construct pBAD33-*gp54*^{Bas11}-*HA*	CAGCGGCTACCCGTATGATGTGCCGGACTATG CATAAAAGCTTGGCTGTTTTGGCG
TZ-10	construct pBAD33-*gp54*^{Bas11}-*HA*	TCATACGGGTAGCCGCTGCTGCCAATAACCTC CATAACAGTATGCTCGTC
TZ-11	generate N275D in *capRel*^{SJ46}	AGATGCAGATCTAAAGAAAAGCCTGCCCAAA AACC
TZ-12	generate N275D in *capRel*^{SJ46}	TTTAGATCTGCATCTGATGTCAGCTTCATTGC
TZ-13	generate D273K in *capRel*^{SJ46}	GACATCAAAAGCAAACCTAAAGAAAAGCCTG CC
TZ-14	generate D273K in *capRel*^{SJ46}	TTTGCTTTTGATGTCAGCTTCATTGCAAATG
TZ-15	generate K278E in *capRel*^{SJ46}	CCTAAAGGAAAGCCTGCCCAAAAACCATAAT GG
TZ-16	generate K278E in *capRel*^{SJ46}	AGGCTTTCCTTTAGGTTTGCATCTGATGTCAG C
TZ-17	generate S279P in *capRel*^{SJ46}	AAAGAAACCGCTGCCCAAAAACCATAATGGA TTC
TZ-18	generate S279P in *capRel*^{SJ46}	GGCAGCGGTTTCTTTAGGTTTGCATCTGATGT C
TZ-23	generate R314E K316E in *capRel*^{SJ46}	TTTTGAAAAAGAAGAATCGGAACAAGCCCTC C
TZ-24	generate R314E K316E in *capRel*^{SJ46}	TCTTCTTTTTCAAAAGGTTTCACAGAAACAAG AAAC
TZ-25	construct pBR322-*capRel*^{SJ46}-*FLAG*	GCGATTACAAGGATGACGATGACAAATAAAT TCCTATGCCTGGCTTAGC
TZ-26	construct pBR322-*capRel*^{SJ46}-*FLAG*	CATCCTTGTAATCGCCGCTGCTGCCCACAGTA TCAATGTGTCTGCTAAGG

TZ-27	*mcp*^{Bas10} into pBAD33	TAAGATTTAAGTATATTAAGGATGACTAAAAAGTATGACGAATTTG
TZ-28	*mcp*^{Bas10} into pBAD33	CTTCTCTCATCCGCCAAAACAGCCAAGCTTTTACACGCCAGTGATTAACACAAG
TZ-29	linearize pBAD33 with added RBS	TATACTTAAATCTTATGGGGCTCGAATTCGCTAGCCCAA
TZ-30	*gp57*^{Bas10} into pBAD33	TACCCGTTTTTTTGGGCTAGCGAATTCGAGATGCAAGACCAGATCGAGGTTAA
TZ-31	*gp57*^{Bas10} into pBAD33	CTTCTCTCATCCGCCAAAACAGCCAAGCTTTTAAATAACCTCCATAACAGTATGCTCGT
TZ-32	*gp60*^{Bas8} into pBAD33	TACCCGTTTTTTTGGGCTAGCGAATTCGAGATGAACTTTGAACAACAAACGCAA
TZ-33	*gp60*^{Bas8} into pBAD33	CTTCTCTCATCCGCCAAAACAGCCAAGCTTTTATTCATCCTCAACAGTTTCAAACACG
TZ-34	*gp57*^{Bas5} into pBAD33	TACCCGTTTTTTTGGGCTAGCGAATTCGAGATGGAAACGCAGGTTGACGTT
TZ-35	*gp57*^{Bas5} into pBAD33	CTTCTCTCATCCGCCAAAACAGCCAAGCTTTTAGATGATCTCCATGTATGTTTCAAAGC
TZ-36	*gp19*$^{SEC\Phi27}$ into pBAD33	TACCCGTTTTTTTGGGCTAGCGAATTCGAGATGGAAAATCAGGTTGATGTTAAAGT
TZ-37	*gp19*$^{SEC\Phi27}$ into pBAD33	CTTCTCTCATCCGCCAAAACAGCCAAGCTTTTAAATGATCTCCATGTATGTTTCAAAGC
TZ-38	generate D6E in *gp54*^{Bas11}	CCAGATAGAAGCTAAAGTTATTCGCCGCAATCC
TZ-39	generate D6E in *gp54*^{Bas11}	TTAGCTTCTATCTGGTCTTGCATCTCGAATTCG
TZ-40	generate A7V in *gp54*^{Bas11}	GATAGACGTGAAAGTTATTCGCCGCAATCC
TZ-41	generate A7V in *gp54*^{Bas11}	ACTTTCACGTCTATCTGGTCTTGCATCTCG
TZ-42	generate I25V in *gp54*^{Bas11}	GAAAGGCGTGGAGATCACAATCGACCTTGAGG
TZ-43	generate I25V in *gp54*^{Bas11}	ATCTCCACGCCTTTCTTGAAGATTCCAGGCG
TZ-44	generate V7A in *gp57*^{Bas10}	GATCGAGGCTAAAGTTATTCGCCGCAATCC
TZ-45	generate V7A in *gp57*^{Bas10}	ACTTTAGCCTCGATCTGGTCTTGCATCTCG
TZ-46	generate V25I in *gp57*^{Bas10}	GAAAGGCATCGAAATCACAATCGACCTTGAGG
TZ-47	generate V25I in *gp57*^{Bas10}	ATTTCGATGCCTTTCTTGAAGATTCCGGG
TZ-48	*capRel*^{SJ46} into pET-His$_6$	ATCACCATCACCATCACGGCAGCGAGAATCTTTATTTTCAGGGCAGCGGCATGGGCAGTGAAGTATATCAAAGCC
TZ-49	*capRel*^{SJ46} into pET-His$_6$	AAGCTGGGTCGGCGCGCCCACCCTTTTACACAGTATCAATGTGTCTGCTAAGGAA

TZ-50	linearize pET-*His_6-capRel*^{SJ46}	AACAATAACAATAACAACAACCCCATGAGCGAGAATCTTTATTTTCAGGGCAGCGG
TZ-51	linearize pET-*His_6-capRel*^{SJ46}	CCAGATTACCAGTTTACCTTCTTCGATTTTCATGCTGCCGTGATGGTGATG
TZ-52	construct pET-*His_6-MBP-capRel*^{SJ46}	ATGAAAATCGAAGAAGGTAAACTGGTAATCTGG
TZ-53	construct pET-*His_6-MBP-capRel*^{SJ46}	GCTCATGGGGTTGTTGTTATTGTT
TZ-54	error-prone PCR insert of *capRel*^{SJ46} C-terminus	GGCAAGCAGCACCGCAAT
TZ-55	error-prone PCR insert of *capRel*^{SJ46} C-terminus	ACATATACATTTGCAATGAAGCTGACATCA
TZ-56	linearize pBR322-*capRel*^{SJ46} for error-prone PCR	ATTGCGGTGCTGCTTGCC
TZ-57	linearize pBR322-*capRel*^{SJ46} for error-prone PCR	TGATGTCAGCTTCATTGCAAATGTATATGT
TZ-58	*gp54*^{Bas11} with native promoter into pBR322	CCTTTCGTCTTCAAGAATTCTCATGTTAGCACTTTTTGTTAAAACCAGTCAGATGC
TZ-59	*gp54*^{Bas11} with native promoter into pBR322	CATCCTTGTAATCGCCGCTGCTGCCAATAACCTCCATAACAGTATGCTCGT
TZ-60	linearize pBR322-*capRel*^{SJ46}*-FLAG*	ACATGAGAATTCTTGAAGACGAAAGG
TZ-61	linearize pBR322-*capRel*^{SJ46}*-FLAG*	GGCAGCAGCGGCGATTAC
