# Supplementary material for: A bacterial immunity protein directly senses two disparate phage proteins
Source: Nature. 2024 Oct 16;635(8039):728–35. doi: 10.1038/s41586-024-08039-y (PMC11578894; doi:10.1038/s41586-024-08039-y)
Supplement: Supplementary file 2 — Reporting summary [file 41586_2024_8039_MOESM2_ESM.pdf]

nature portfolio

Corresponding author(s): Garcia-Pino, Laub

Last updated by author(s): Aug 2, 2024

Reporting Summary

Nature Portfolio wishes to improve the reproducibility of the work that we publish. This form provides structure for consistency and transparency in reporting. For further information on Nature Portfolio policies, see our Editorial Policies and the Editorial Policy Checklist.

Statistics

For all statistical analyses, confirm that the following items are present in the figure legend, table legend, main text, or Methods section.

n/a	Confirmed	
☐	☒	The exact sample size (*n*) for each experimental group/condition, given as a discrete number and unit of measurement
☐	☒	A statement on whether measurements were taken from distinct samples or whether the same sample was measured repeatedly
☐	☒	The statistical test(s) used AND whether they are one- or two-sided<br>*Only common tests should be described solely by name; describe more complex techniques in the Methods section.*
☒	☐	A description of all covariates tested
☒	☐	A description of any assumptions or corrections, such as tests of normality and adjustment for multiple comparisons
☐	☒	A full description of the statistical parameters including central tendency (e.g. means) or other basic estimates (e.g. regression coefficient) AND variation (e.g. standard deviation) or associated estimates of uncertainty (e.g. confidence intervals)
☐	☒	For null hypothesis testing, the test statistic (e.g. *F*, *t*, *r*) with confidence intervals, effect sizes, degrees of freedom and *P* value noted<br>*Give P values as exact values whenever suitable.*
☒	☐	For Bayesian analysis, information on the choice of priors and Markov chain Monte Carlo settings
☒	☐	For hierarchical and complex designs, identification of the appropriate level for tests and full reporting of outcomes
☒	☐	Estimates of effect sizes (e.g. Cohen's *d*, Pearson's *r*), indicating how they were calculated

Our web collection on statistics for biologists contains articles on many of the points above.

Software and code

Policy information about availability of computer code

Data collection	NCBI BLAST for homology searches.<br>Consurf 2016 web server for identifying additional homologs.<br>XDS suite: Jun 30, 2023 (BUILT 20230630)
Data analysis	Coot: 0.9.8.93 for crystallography.<br>Phenix: 1.20.1-4487 for crystallography.<br>Buster/TNT: 2.10.4 for crystallography.<br>UCSF ChimeraX version 1.7rc202311230013 for structural visualization.<br>Geneious 2022.0.2 for genome and sequence alignment.

For manuscripts utilizing custom algorithms or software that are central to the research but not yet described in published literature, software must be made available to editors and reviewers. We strongly encourage code deposition in a community repository (e.g. GitHub). See the Nature Portfolio guidelines for submitting code & software for further information.

Data

Policy information about availability of data

All manuscripts must include a data availability statement. This statement should provide the following information, where applicable:

- Accession codes, unique identifiers, or web links for publicly available datasets
- A description of any restrictions on data availability
- For clinical datasets or third party data, please ensure that the statement adheres to our policy

Structural data for Gp54 and the CapRelSJ46-Gp54 complex are available in PDB (9AXB and 9ERV, respectively). Sequencing data are available in the Sequence Read Archive (SRA) under BioProject PRJNA1084025. HDX-MS data can be accessed via ProteomeXchange with identifier PXD050526. AlphaFold predicted structural models are deposited in the ModelArchive Database (https://www.modelarchive.org) with the accession codes ma-zblch (DOI:10.5452/ma-zblch) and ma-9z23e (DOI:10.5452/ma-9z23e). All other data are available in the manuscript or the supplementary materials. Other previously published structures are available in PDB (7ZTB, 5LD2). UniRef90 database is publicly available. Reference phage genomes are publicly available: SECΦ27 (NC_047938.1), Bas05 (MZ501101.1), Bas08 (MZ501059.1), Bas10 (MZ501077.1), Bas11 (MZ501085.1). Materials including strains and plasmids are available upon reasonable request.

Research involving human participants, their data, or biological material

Policy information about studies with human participants or human data. See also policy information about sex, gender (identity/presentation), and sexual orientation and race, ethnicity and racism.

Reporting on sex and gender	N/A
Reporting on race, ethnicity, or other socially relevant groupings	N/A
Population characteristics	N/A
Recruitment	N/A
Ethics oversight	N/A

Note that full information on the approval of the study protocol must also be provided in the manuscript.

Field-specific reporting

Please select the one below that is the best fit for your research. If you are not sure, read the appropriate sections before making your selection.

☒ Life sciences ☐ Behavioural & social sciences ☐ Ecological, evolutionary & environmental sciences

For a reference copy of the document with all sections, see nature.com/documents/nr-reporting-summary-flat.pdf

Life sciences study design

All studies must disclose on these points even when the disclosure is negative.

Sample size	No sample size calculation was performed. Sample sizes were chosen based on the number needed to reliably determine differences between groups. Given large effect sizes, we chose to replicate experiments 2-3 times as is routine to simply indicate reproducibility.
Data exclusions	No data were excluded from analysis.
Replication	All experimental findings were repeated at least twice. All reported results were successfully reproduced.
Randomization	No experimental groups or control groups were subjectively chosen and there are no covariates to control for as experiments were done in isogenic strains. No randomization is required.
Blinding	Blinding was not required because all data were obtained objectively and had strong effect sizes over multiple independent replicates and raw data are reported in the manuscript.

Reporting for specific materials, systems and methods

We require information from authors about some types of materials, experimental systems and methods used in many studies. Here, indicate whether each material, system or method listed is relevant to your study. If you are not sure if a list item applies to your research, read the appropriate section before selecting a response.

Materials & experimental systems

n/a	Involved in the study
☐	☒ Antibodies
☒	☐ Eukaryotic cell lines
☒	☐ Palaeontology and archaeology
☒	☐ Animals and other organisms
☒	☐ Clinical data
☒	☐ Dual use research of concern
☒	☐ Plants

Methods

n/a	Involved in the study
☒	☐ ChIP-seq
☒	☐ Flow cytometry
☒	☐ MRI-based neuroimaging

Antibodies

Antibodies used

HA-tag (C29F4) rabbit mAb (Cell Signaling Technology #3724)
(FLAG) DYKDDDDK Tag (D6W5B) Rabbit mAb (Cell Signaling Technology, #14793)
Anti-RpoA antibody (Biolegend Cat#: 663104).

Validation

Antibodies have been validated based on manufacturer's website:
anti-HA: validated by "western blot analysis of extracts from HeLa cells, untransfected or transfected with either HA-FoxO4 or HA-Akt3".
anti-FLAG: validated by "western blot analysis of extracts from 293T cells, mock transfected (-) or transfected with DYKDDDDK-GFP".
In addition, all western blotting experiments include controls with untagged proteins as internal validation for antibodies.
anti-RpoA: validated by western blots using "total lysates from HeLa and E.coli BL21 cells" by the manufacturer, and widely used in bacterial studies.
All antibodies were used according to the manufacturer's guidelines.

Plants

Seed stocks

N/A

Novel plant genotypes

N/A

Authentication

N/A
